# Correction

**DOI:** 10.1080/13880209.2023.2233815

**Published:** 2023-07-13

**Article title**: Phosphatidylinositide 3-kinase inhibition: A new potential target for the treatment of polycystic ovarian syndrome

**Authors:** Shah, K. N. & Patel, S. S.

**Journal**: *Pharmaceutical Biology*

**Bibliometrics**: Volume 54, Number 6, pages 975–983

**DOI**: https://doi.org/10.3109/13880209.2015.1091482

In the version of this article initially published, there was a mistake in the images of normal control groups of Figures 2 and 3.

Figures 2 and 3 are now corrected and the article has been republished online.

